# Self-organized sorting limits behavioral variability in swarms

**DOI:** 10.1038/srep31808

**Published:** 2016-08-23

**Authors:** Katherine Copenhagen, David A. Quint, Ajay Gopinathan

**Affiliations:** 1 University of California Merced, Merced CA, USA; 2 Stanford University, Stanford CA, USA; 3 Carnegie Institute of Washington, Stanford CA, USA

## Abstract

Swarming is a phenomenon where collective motion arises from simple local interactions between typically identical individuals. Here, we investigate the effects of variability in behavior among the agents in finite swarms with both alignment and cohesive interactions. We show that swarming is abolished above a critical fraction of non-aligners who do not participate in alignment. In certain regimes, however, swarms above the critical threshold can dynamically reorganize and sort out excess non-aligners to maintain the average fraction close to the critical value. This persists even in swarms with a distribution of alignment interactions, suggesting a simple, robust and efficient mechanism that allows heterogeneously mixed populations to naturally regulate their composition and remain in a collective swarming state or even differentiate among behavioral phenotypes. We show that, for evolving swarms, this self-organized sorting behavior can couple to the evolutionary dynamics leading to new evolutionarily stable equilibrium populations set by the physical swarm parameters.

Collective motion in natural systems is a well studied phenomena that spans many spatial and temporal time scales^1^,^2^, ranging from protein filaments driven by molecular motors^3^,^4^, to swarming bacteria^5^ and active colloidal crystals^6^, to fish^7^, birds^8^, and even robot swarms^9^,^10^. Such collective phenomena rely on the sharing or transmission of local information by constituents of the group leading to global consensus. Information flow and information fidelity are therefore critically important for collective systems to persist, especially in situations where disorder is an issue.

Disorder may exist in the environment and it has been recently shown that topological disorder in the form of obstacles can have significant detrimental effects on the ability for systems to move collectively^11^,^12^,^13^. Disorder can also exist in the form of variability in behavior among members of the group, which has been modeled using agents with varying velocities^14^,^15^ or differing noise sensitivity^16^, agents that stochastically switch off social interactions^17^ and migratory groups of agents with varying sensitivity to external directional cues^18^,^19^. An interesting manifestation of such behavioral heterogeneity would be swarms with agents that have varying abilities to process social cues. A specific scenario could be swarms with sub-populations of non-aligners that either refuse or are unable to participate in utilizing shared information with their neighbors resulting in significant impacts on the collective. For example, it has been observed in colonies of the quorum sensing bacteria *P. aureginosa* that colony collapse can occur if mutant cheaters (those that do not secrete signaling compounds to sustain colony swarming) are introduced into the swarm even though there are sufficient nutrients in the environment^20^,^21^,^22^,^23^,^24^. Differential behavior can also be a result of disease in organisms. For example, in locust swarms, the introduction of parasites (Paranosema locustae) inhibit the production of aggregation pheromones that promote gregarious behavior, thus resulting in a transition to a non-swarming state^25^. In analogy to these biological systems, non-alignment behaviors could also arise in robotic drone swarms^9^,^26^, where software viruses or hardware failures could cause agents to malfunction, leading to an inability to swarm effectively and requiring the development of novel algorithms to alleviate this problem^27^,^28^,^29^.

Since differential behavior or non-alignment can be fairly ubiquitous in swarming systems and too many such non-aligners will have a deleterious effect on the ability to swarm, it poses a number of significant questions regarding how natural swarms deal with non-aligners or more generally variations in alignment ability. Is there a maximum fraction of non-aligners that a finite swarm can carry and is there a simple predictive relation between the maximum non-aligner carrying capacity and the characteristics of the swarm? Could there exist robust mechanisms that allow the whole group to increase its non-aligner carrying capacity? Are there mechanisms that allow swarms to limit the spread of non-aligning? Do these same mechanisms operate if the alignment abilities have a more natural continuous spread? If there are fitness advantages associated with non-aligning how does it affect the evolutionary dynamics? To address these issues, we study an agent based model of finite swarms with local aligning and cohesive interactions between neighbors and a sub-population of non-aligning agents within the group that do not align with their neighbors and do not have a defined internal preferred velocity.

## The Model

Agents exist in a continuous two dimensional space where each agent interacts with other agents in its immediate neighborhood defined by a vision radius *d*_*v*_. At any given time, *t*, each agent has a position 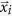, and a direction of travel 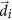. The direction of travel of the agent at the next time step is computed by taking into account both alignment and cohesive interactions with its neighbors as well as noise. This is implemented by computing an alignment interaction vector, 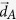 and a cohesive interaction vector, 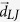. Here, 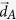 for an agent is the sum of the direction vectors of its nearby neighbors, which is proportional to the mean consensus direction (see methods for details). The form of the alignment interaction we use is common in studies of swarming systems and can be found in many variants of the standard Vicsek model^30^,^31^,^32^, which was formulated originally to study a thermodynamic phase transition of aligning agents resulting from an increase in temperature or noise of the system. The form of the cohesive interaction is modeled as arising from a Lennard-Jones (LJ) interaction which is attractive at large distances and repulsive at close range (see methods for details). The effect of the LJ interaction in our model is to encourage agents to maintain a preferred distance (*R*) thus providing an overall cohesiveness as well as collision avoidance within the swarm, as seen in similar swarming systems^29^,^33^,^34^. It is to be noted that our results do not depend critically on the exact form of the potential as long as it prescribes a preferred separation distance. In what follows, we set 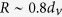, so that each agent sees only its first nearest neighbors on average, consistent with prior flocking models. The updated travel direction vector is then
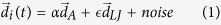


Noise here is implemented as an angular adjustment by rotating the calculated travel direction vector through a randomly selected angle from a uniform distribution in the interval [−*η*/2, +*η*/2]. This is described in detail in the methods section below. Here the dimensionless parameters *α* and 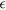 measure the importance of alignment and cohesiveness respectively (equation (1)). Directional updates are only performed in time intervals of *δt* corresponding to the physical reaction time of agents within the system. In contrast, positions are updated every simulation time-step, Δ*t*, which can be made arbitrarily small. It is to be noted that velocity is encoded by a new length scale, *d*_*r*_, the reaction distance, which determines how far an agent moves before reprocessing information, i.e. in time *δt*. Our model for the swarm produced expected stable collective dynamics^30^, including order-disorder transitions as a function of noise (see Supplementary Information 1.1), which have been subsequently studied using both hydrodynamics^35^,^36^, and agent based models^37^. Although such agent based models are quite simple and leave out many microscopic details, they have been shown to produce a rich and complex phase space of collective motion that mimics the behavior of real physical and biological swarming systems^37^,^38^,^39^,^40^.

To introduce behavioral heterogeneity in the form of non-aligners who refuse, or are unable, to participate in utilizing local alignment cues to guide their movements, we choose, at random, a fraction *f* of the swarming agents to be non-aligners after a transient time period where collective motion is established. Non-aligner agents follow the same update procedures as aligners, except that the alignment interaction is suppressed by setting *α* = 0, so they no longer have any intrinsic tendency to align with their neighbors. Thus the only forces the non-aligners feel arise from the LJ potential.

## Results

### Large inter-agent cohesiveness limit

**Figure 1:**
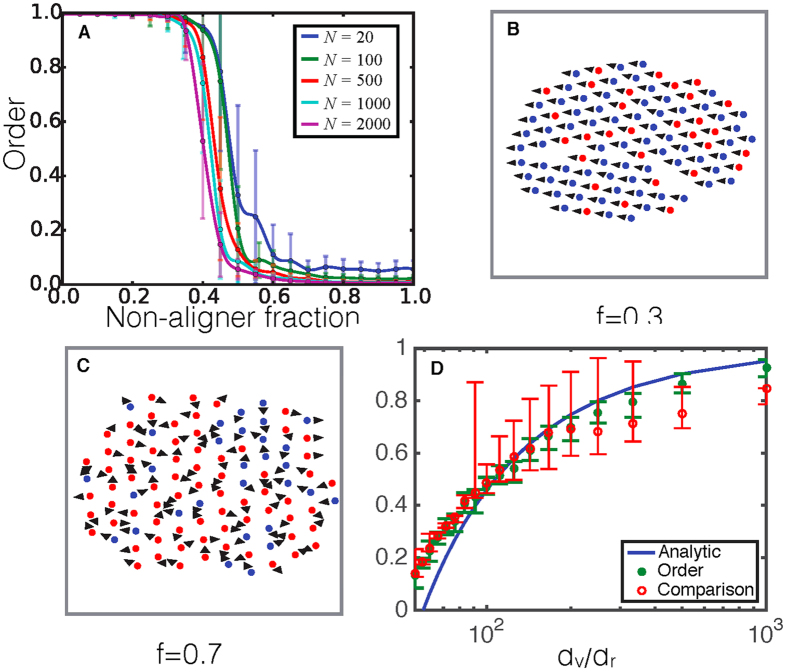
(**a**) Order vs non-aligner fraction of swarms with varying system sizes, 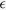 = 10^0^. There is a clear transition from an ordered state with 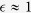 to a disordered state with low 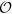 at non-aligner fractions above a critical value, f*. (**b**) Simulation snapshot with a value of *f* below the critical value, the agents (aligners shown in blue and non-aligners in red) all travel in the same direction (black arrow heads). (**c**) When the non-aligner fraction is larger than the critical value, the agents try to move in different directions, resulting in a net zero polarization. (**d**) f* for the order-disorder transition (green) as a function of *d*_*v*_/*d*_*r*_ compared to the non-aligner fraction at which the measured average cohesion becomes greater than that of the average alignment (red). The blue curve is the result of our analytical approximation comparing average alignment and cohesiveness.

We first consider the limit where the cohesive interactions are strong enough to produce a single swarm that cannot break apart and shows no rearrangements over time. This happens when 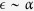, which is high enough to prevent the swarm from fracturing. Thus, once a random spatial distribution of non-aligners is established in the swarm, it remains fixed in a *locked-in* state thereafter. We begin by focusing on the effects of adding a fixed fraction of non-aligners, *f*, to our system and analyzing how the addition of these non-aligners affects the ability of the agents to swarm. To quantify the degree of order in the swarm we use the group polarization of all the agents, 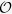, defined by,
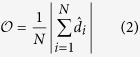


Figure 1(a) shows that as *f* increases, the swarm undergoes a sharp transition from an ordered swarming state where 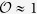 to a static disordered non-swarming state with 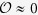. The transition occurs at a critical non-aligner fraction, f*, that is very weakly dependent on the system size as seen in Fig. 1(a) where the transition occurs near f* ∼ 0.5 for 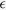 = 1, *d*_*v*_/*d*_*r*_ = 100, and various different system sizes. On the left of the transition the system has a low non-aligner fraction and all agent directions are aligned with each other, shown by the black arrow heads in Fig. 1(b) with non-aligners in red and aligners in blue, while on the right of the transition the system is no longer able to form an ordered state, and agents instantaneously have random directions (Fig. 1(c)), resulting in a state with no net movement of the center of mass of the swarm. Note however that each individual still moves within the cluster even in the disordered state. It is to be noted that the time at which non-aligners are introduced does not affect the results shown and there appears to be very little hysteresis in the system (see Supplementary Information 1.2 and 1.3), suggesting a possible first order transition^17^, which implies that the results for a fixed non-aligner fraction are fairly unique. Intuitively, increasing the fraction of non-aligners, *f*, while keeping the swarm together as a single unit, decreases net alignment with different parts of the swarm trying to go in different directions and results in a disordered stationary state. With cohesive strength being fixed at a high value, the non-aligner carrying capacity, f*, then depends sensitively on the ratio, *d*_*v*_/*d*_*r*_, where *d*_*v*_ ∼ *R* is the vision radius or agent-spacing and *d*_*r*_ is the reaction distance. Figure 1(d) shows the value of f* where the system transitions from an ordered to a disordered state (green) increasing with the ratio *d*_*v*_/*d*_*r*_.

To identify the cause for this behavior, we looked at the average magnitude of the alignment interaction, 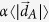, over all agents in the swarm, and the average magnitude of the cohesive interaction, 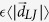. As the non-aligner fraction *f* increases, we found that the average magnitude of the alignment interaction, 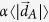 decreases as the non-aligners do not contribute to alignment and that the transition occurs at a value of *f* ∼ f* when 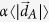, becomes less than 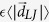. Figure 1(d) shows the value of *f* where the measured value of 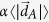 in the simulation becomes less than 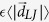 (red) and we see that it compares well with the measured value f* for the order disorder transition. Comparing the alignment and cohesive interactions also allows us to derive a simple analytic approximation for the value of f* with no adjustable parameters (Equation 3).
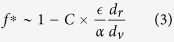
here, *C* is a constant set by the form of the cohesive interaction as well as the average number of neighbors per agent (See Supplementary Information 2 for derivation). The agreement between our analytical estimate and the simulation values is shown by the blue curve in Fig. 1(d).

The fact that f* changes from about 10% to about 90% in just over a decade in the parameter *d*_*v*_/*d*_*r*_ is of significance. Higher values of *d*_*v*_/*d*_*r*_ correspond to situations where agents are spaced much further than they move between updating directions, resulting in agents being able to quickly correct for deviations from their perfect swarming formation. Thus a potential way for swarms to accommodate more non-aligners or malfunctioning agents is to increase *d*_*v*_/*d*_*r*_, which can be accomplished either by increasing their separation (increases *d*_*v*_) or by moving slower and/or processing information more quickly (decreases *d*_*r*_). The other mechanism to potentially increase the non-aligner carrying capacity f*, is by reducing the cohesiveness 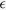/*α*.

### Tuning cohesiveness between agents allows for enhanced swarming

**Figure 2:**
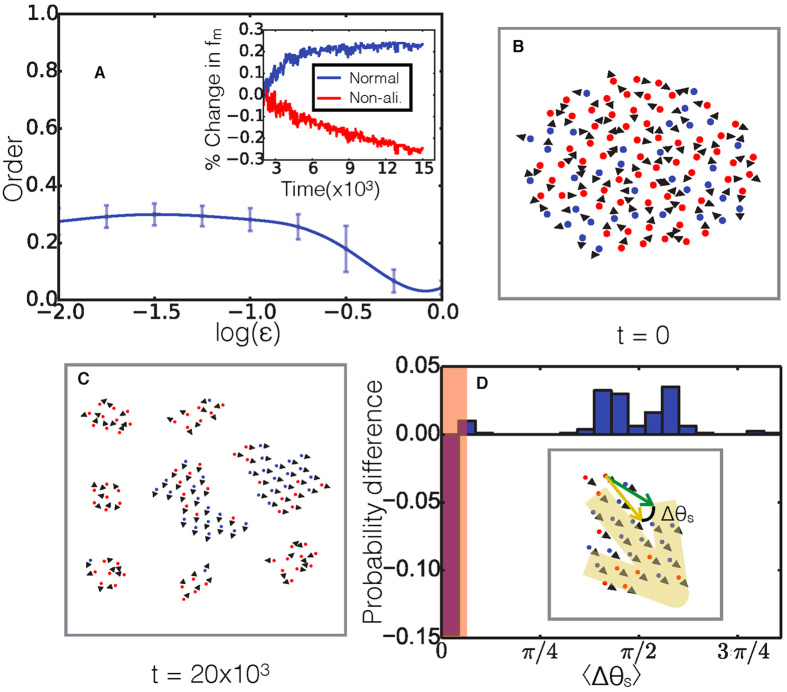
(**A**) The weighted average steady state order of all clusters against 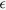, for a fixed value of non-aligner fraction, *f* = 0.8 and system size of *N* = 100. (A inset) Plot of the percent change in mobility fraction for aligners and non-aligners against time after the initial transient phase with fixed 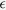 = 10^−1^ and *f* = 0.8. (**B**) At values of non-aligner fractions near the transition the system sorts from one single swarm without any order, to (**C**) smaller clusters with ordered clusters and disordered clusters. Separation shown between clusters in (**C**) is chosen for convenience and is much smaller than the actual separations by the end of the simulation. (**D**) Difference in the probability distribution of the angle between the direction of travel of non-aligners from the average cluster direction and the direction of aligners from the average cluster direction. Red shaded region is the angles which are within the range of the noise of the system.

Reducing the cohesiveness results in a situation where the disorder is no longer quenched and the agents are able to rearrange themselves within the swarm or even cause the swarm to fracture. To study these cases, the system is initialized with a high 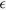 = 1 in the *locked-in* state and a high value of *f* > f* so that the system is in a static disordered state by default. After a transient time period allowing the system to reach a steady state, 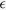 is reduced, allowing for potential rearrangements and fracture. When 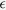 is reduced, we found that the system fragments into smaller clusters which can individually be ordered swarming clusters or disordered static clusters. Figure 2(A) shows the weighted average of the steady state order parameter 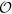 of the fragmented clusters of the system for a fixed non-aligner fraction *f* = 0.8 versus the relative strength of the LJ interaction, 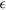, for a system size of *N* = 100. A modest reduction in 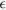 appears to have a dramatic effect. When 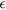 = 10^−1.0^ it appears that the polarization in the swarm is greatly amplified where swarming was completely suppressed in the quenched configuration case 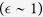. Thus, the previously immobile single cluster has now broken up into smaller clusters, many of which are mobile. As 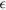 is decreased further, the polarization begins to decay slightly suggesting that the cohesiveness has become so weak that some of the clusters begin to fracture into individual agents and thus are no longer part of ordered clusters. This suggests that there is some optimal regime of 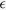 for collective motion in the presence of non-aligners.

We next probed the dynamics associated with the cluster reorganization. Figure 2(A inset) shows the percent change in the fraction of aligners (blue) and non-aligners (green) which are part of moving clusters as the simulation time progresses. The mobility fraction of non-aligners steadily decreases while the mobility fraction of aligners steadily increases. This suggests a sorting mechanism whereby non-aligners are being left behind allowing for clusters with more aligners to become ordered and move collectively. This can be visualized in Fig. 2(B,C) which illustrates how the system starts out as a single disordered group and then breaks apart into smaller ordered clusters with mixtures of non-aligners and aligners, as well as disordered clusters consisting of mostly non-aligners.

To examine the relative contribution of rearrangements within single clusters to the overall sorting, we measured the probability distribution of the angle between the direction of travel of the non-aligners and the cluster average, as well as the same probability distribution for aligners. We then calculated the difference in these two probability distributions (Fig. 2(D)). We observe that aligners contribute predominantly to angles close to zero (purple bar) within the spread due to noise (red shaded region), while non-aligners are more likely to travel at angles around *π*/2 (blue bars) with respect to the cluster average. This implies that non-aligners will gradually move towards the sides and backs of the clusters before getting left behind. By sorting out non-aligners, the over all average alignment interaction strength in the cluster increases due to the higher fraction of aligners present, and the smaller cluster is able to swarm.

**Figure 3:**
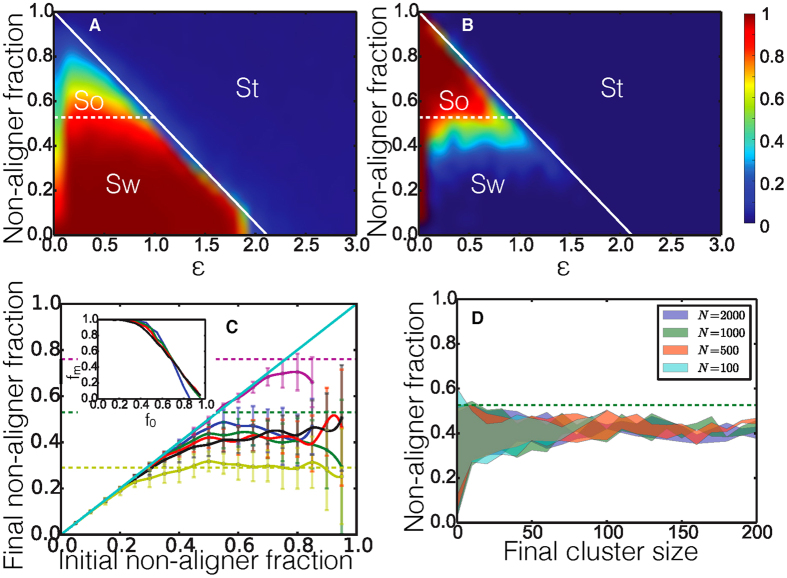
(**A**) The order parameter of the system with *d*_*v*_/*d*_*r*_ = 100 plotted by color against 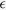 and *f*. (**B**) The probability of the system fracturing into two or more clusters plotted against 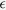 and *f*. The three phases, swarming (Sw), sorting (So), and static (St) are labeled on the phase diagram, with the predicted ordering transition (from equation (3)) as a solid white line and the dashed white line corresponding to the critical non-aligner fraction at 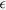 = 1. (**C**) The non-aligner fraction within small clusters which are ordered in steady state vs. the initial non-aligner fraction *f*, with *N* = 100, and 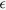 = 10^−0.5^,10^−1.0^,10^−1.5^, and 10^−2.0^, corresponding to the blue, green, red, and black curves respectively. The dashed green line shows the value of f* = 0.5 with *d*_*v*_/*d*_*r*_ = 100. The purple curve corresponds to f* = 0.8, purple horizontal dashed line, with *d*_*v*_/*d*_*r*_ = 200, and 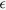 = 10^−1.0^. The yellow curve corresponds to f* = 0.2, yellow horizontal dashed line, with *d*_*v*_/*d*_*r*_ = 67, and 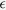 = 10^−1.0^. The light blue line shows the non-aligner fraction in the system if it were unable to fracture. (C inset) The fraction of the system that is in ordered clusters for *N* = 500. (**D**) The spread of non-aligner fractions within the sorted swarming clusters for several different systems sizes. The shaded area shown for each system size represents the average ± one standard deviation, for final cluster sizes in bins of size 10. Green dashed line as in (**C**).

We now consider the full range of behaviors exhibited by our system as the parameters, including cohesion and non-aligner fraction, are varied. Figure 3 (A) shows a phase diagram of the system in the non-aligner fraction/cohesion (*f*,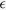) plane where the colors indicate the value of the order parameter 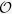. We see that if the non-aligner fraction exceeds a critical value f*(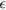) predicted by equation (3) (solid white line), the system is in a disordered, *static* state - denoted “St”. Here, the cohesive forces dominate over the alignment, leading to the static state with no net order, due to the frustration of agents attempting to travel in differing directions exceeding a value which can be resolved by aligning to reach a consensus direction. Below the line, we see that the system is able to achieve order. There are, however, two very different behaviors within this region. For low values of *f*, the system is stable and achieves a high degree of polarization (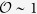) as a *single* cohesive swarm, relieving frustration by aligning to reach a consensus direction. This is clearly shown by the *swarming* region labelled “Sw” in Fig. 3(B) where the colors now indicate the probability that the system fractures into two or more clusters. Now, as the non-aligner fraction is increased, we enter the sorting regime (“So”) that we examined earlier in this section, where the system begins to fracture into smaller clusters, leaving behind non-aligner rich clusters, allowing aligner rich clusters to swarm and thereby increasing the net polarization. It is of interest to note here that the transition between the swarming and sorting phases roughly occurs at a constant value of the non-aligner fraction, *f*^*c*^ equal to the critical non-aligner fraction, f*, when the alignment and cohesion constants become equal, 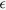 = 1 (in units of *α*), which is shown by the dashed white line, suggesting that at non-aligner fractions above *f*^*c*^ frustration within the system is relieved by fracture as well as alignment. The existence of these three regimes and their locations relative to the predicted transition lines turn out to be completely general and work over the range values of *d*_*v*_/*d*_*r*_ feasible in our simulations with no adjustable parameters (see Supplementary Information 3).

### Self-organized sorting

Starting with a cluster with a high non-aligner fraction, we showed that, for low enough cohesive energies, the swarm dynamics spontaneously cause the re-organization and break-up of the cluster. What, then, is the non-aligner fraction of the sub-clusters that are now mobile and able to swarm? To answer this question, we looked more closely at the fragmented clusters and measured the average non-aligner fraction of all swarming clusters which have 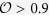. Figure 3(C) shows the final fraction of non-aligners averaged over all swarming sub-clusters as a function of the initial non-aligner fraction in the system, for three different values of *f*^*c*^ of ∼0.3, 0.52 and 0.75, which is set by changing the value of *d*_*v*_/*d*_*r*_ according to Fig. 3(D). For low values of the initial non-aligner fraction, *f*, the final non-aligner fraction appears to follow linearly with a slope of unity, implying that little sorting or fracturing occurs for low non-aligner fractions. This is as expected, since for non-aligner fractions below the transition for a single crystallized swarm, at *f*^*c*^, are within the “Sw” region of the phase diagram in Fig. 3(A,B), and no fracturing is necessary to relieve frustrations within the system.

Then, as the initial non-aligner fraction continues to increase and approach *f* ≈ *f*^*c*^, we enter a region where the final non-aligner fraction seems to level off. In this regime, the value of *f* is high enough that, if the cohesiveness, 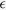, were very high (>1), the system would be in a frustrated state with no overall alignment and different agents trying to go in different directions but being held together. But since the cohesiveness is actually low, the frustration can be relieved by the cluster fragmenting into smaller pieces that can have a net alignment. The same logic holds for resulting smaller clusters that still have too high of a non-aligner fraction *f* - they will continue to fragment. If a resulting cluster has a non-aligner fraction less than ∼*f*^*c*^, it is within the “Sw” region of the phase diagram in Fig. 3(A,B), so it can move collectively by reaching a consensus direction to relieve frustrations without fragmenting further. Thus the aligners have effectively self-organized into small clusters with an average non-aligner fraction close to the critical value *f*^*c*^. This picture is confirmed by Fig. 3(C inset) which shows the fraction of the system which is contained in ordered moving clusters. For low initial non-aligner fractions, almost the entire cluster is mobile, while beyond the critical non-aligner fraction, the mobility fraction decays linearly as predicted by the simple picture where non-aligners in excess of the critical fraction *f*^*c*^ are simply left behind.

To examine how robust our results were, and, in particular, if they were sensitive to the initial system size, we fixed 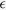 and non-aligner fraction at 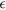 = 10^−1.0^, and *f* = 0.8, and examined the final swarming clusters for a range of initial system sizes. We also measured the spread of non-aligner fractions within final clusters of different sizes, again for a few systems sizes. Figure 3(D) shows the average ± standard-deviation of the non-aligner fraction over the different final cluster sizes. We can see that both the average non-aligner fraction and its variance within the final clusters are fairly independent of initial system size.

One can now imagine a generic swarm where the fraction of non-aligners (or defective/diseased agents) increases over time. This could be due to the spreading of a disease among organisms or the breakdown/malfunctioning over time of individual robotic agents, or simply the tendency of entropy to increase. As time goes on, the system will reach a state where the fraction of non-aligners exceeds the critical fraction, *f*^*c*^, thus entering the sorting regime, if the cohesive forces are in the right regime. Our results indicate that the system can then shed almost the entire excess non-aligner population (above the critical threshold) and revert to a fraction close to but just below *f*^*c*^. Then as the system evolves again over time the non-aligner fraction increases past the threshold and the process begins again. Thus, dynamical sorting can compete against natural processes that drive increases in the non-aligner fraction and provide a mechanism for the system to reach a dynamic equilibrium, regulating its composition so that it sits at the edge of criticality in behavior; reminiscent of a self-organized critical system.

### Swarms with a distribution of alignment behaviors

So far, we have considered behavioral variability to be binary in character with agents being either aligning or non-aligning (*α* = 1, 0). It is, however, quite reasonable to expect that many naturally occurring swarms might display a spectrum of behaviors that corresponds to natural abilities differing from individual to individual. We showed before that the phase behavior of the swarm is controlled by the mean level of alignment as compared to cohesion. Here we consider what happens if, instead of changing the mean level of alignment accomplished by changing the fraction of non-aligners in a binary system, we keep the mean value fixed and change the spread in a system with continuous values of the alignment interaction strength *α*. To implement this, we selected *α* for each agent from a Gaussian distribution with mean, *μ*, and standard deviation, *σ*. We consider two types of systems, one with a high value of *μ* that would result in an ordered swarming system in the *σ* = 0 case and one with a low value of *μ* which would result in a static system with *σ* = 0. Keeping these two values of *μ* fixed, we vary the spread *σ*.

**Figure 4:**
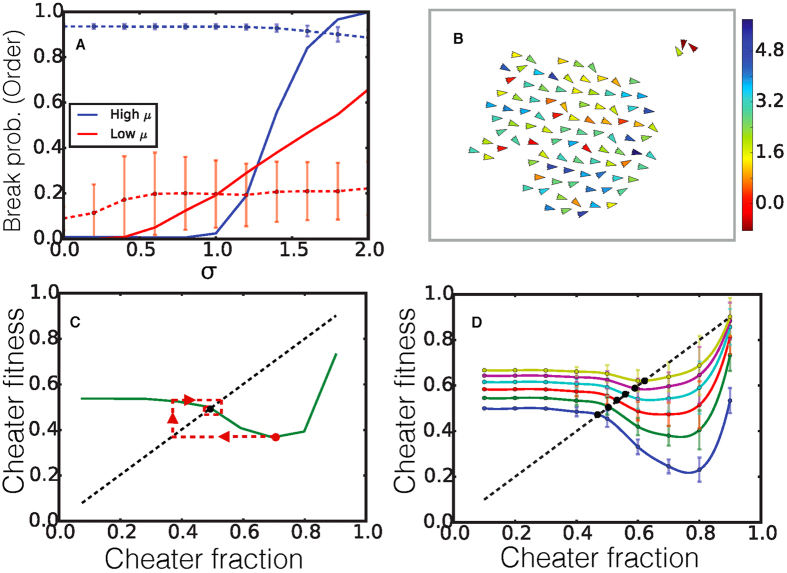
(**A**) Probability of fracture (solid), and order parameter (dashed) plotted against *σ* for a high value of *μ* = 2.5(blue) and a low value of *μ* = 0.5(red). (**B**) Snapshot from a simulation with *μ* = 2.5, and *σ* = 1.2 showing fragments of a larger cluster that has sorted itself into an ordered cluster with a higher mean *α* and static, disordered cluster with *α* ∼ 0 for most agents. Arrowheads signify direction of motion, and the color bar shows the value of *α* for each individual agent. (**C**) The relative fitness of all cheaters, 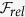, as a function of cheater fraction, *f* for Φ = 0.2. The point where the dashed diagonal line crosses the fitness curve is a fixed point where the cheater fraction and fitness of the system become equal representing an equilibrium fixed point for the evolutionary dynamics. Red dashed line shows an example trajectory an evolving system would follow starting at *f* = 0.7 and approaching the fixed point after several generations. (**D**) The relative fitness of all cheaters, 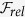, in the system as a function of cheater fraction for different values of the ratio of fitness advantage constants 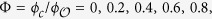 and 1 corresponding to the blue, green, red, cyan, purple and yellow lines respectively. The points were the dashed diagonal line crosses the fitness curves are evolutionary fixed points. For this system *N* = 500, *d*_*v*_/*d*_*r*_ = 100, and 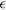 = 10^−0.5^.

Figure 4(A) shows both the low *μ* (blue) and high *μ* (red) cases and we can see that as *σ* increases there exists a critical threshold, *σ*_*c*_, beyond which the system will be likely to fracture (solid lines show probability of fracture), however the reason for fracture is different in both cases. The average order parameter (dashed line) for the high *μ* case starts out high and as *σ* increases and fracture begins to occur, the order decreases slightly because some individuals now have a low enough *α* that they behave like non-aligners and break off of the main body of swarmers to form static clusters. In the case where *μ* is low the system has very low order at low *σ* and as the spread of *α* values increases the system begins to fracture and the average order increases. In this case some agents are reaching high enough *α* values to form an ordered state and will create clusters of ordered moving agents which break off from or fragment the static main body of agents. Figure 4(B) shows a snapshot from the simulation that started off as one swarming cluster. Here *σ* is high enough to allow fracture resulting in the sorting out of a smaller, static cluster of agents with a predominantly low value of *α*. Thus the increase in the spread of *α* has similar consequences whether one starts with a high or low average *α*. There is a self-organized separation of the population leading to clusters of similar “phenotype” providing a mechanism for a natural limitation to the extent of variability in any swarming population.

### Evolving populations

We now examine the idea that dynamical sorting could be a mechanism for *evolving* swarms to robustly sustain a certain composition over longer timescales. For swarms, over sufficiently long timescales, one could imagine that the non-aligner population fraction increases due to some fitness advantage that the non-aligners derive by not participating in orientational information processing. For example, in ref. 18, an agent’s ability to decide on a preferred travel direction due to social interactions or migratory tendency is assumed to come at some cost due to energy expenditure or reduced predator awareness during directional information processing. In this case the non-aligners would be cheaters in the evolutionary sense which can lead to some interesting dynamics. To model the evolutionary dynamics we considered a model replicator system^41^ where agents replicate themselves at a rate that is determined by their fitness, with the fitter species (cheater or non-cheater) being able to reproduce more quickly and increasing its proportion in the population over time. The assigned fitness of an individual agent depends on whether an agent is a cheater and whether it is in a swarming cluster. We take the fitness of an agent *i* at any point in time to be given by,
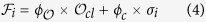
here, 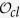 is the polarization of the cluster that agent *i* belongs to, 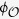 represents the fitness advantage that agents gain by being in ordered clusters and *ϕ*_*c*_ is the fitness advantage gained by cheating agents (say by avoiding energy expenditures associated with orientational processing or signaling) and *σ*_*i*_ is equated to 0 for non-cheaters and 1 for cheaters. These fitness advantages are similar qualitatively to those postulated to study evolution in migratory populations where there were costs associated with obtaining directional cues from the environment and an overall fitness associated with migrating close to the desired direction^18^,^42^,^43^. We start with a system of *N* = 500 agents with 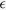 = 10^−0.5^ with periodic boundary conditions at different total cheater fractions. For each simulation, the system is allowed to evolve until a steady state is reached and the relative fitness of all cheaters, 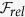, given by
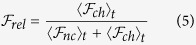
is measured. Here 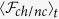 is the time averaged fitness of all cheaters/non-cheaters given by Eq 4. As an example, Fig. 4(C) shows how the relative fitness of cheaters 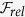 varies with cheater fraction for the ratio of fitness advantage constants 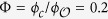. In our evolutionary dynamics, we take the relative fitness of all cheaters, 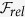, to be the expected fraction of cheaters in the subsequent generation. The red dashed line in Fig. 4(C) shows a typical evolutionary trajectory. Here, a population that starts with a cheater fraction *f* = 0.7 has 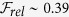 which results in a cheater fraction *f* ∼ 0.39 in the subsequent generation which has 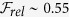 and so on. A fixed point at which the initial cheater fraction is equal to that in the next generation is then given by the intersection between the curve and the diagonal (black dot in Fig. 4(C)). An evolutionarily stable fixed point is obtained when a fixed point is an attractor of all evolutionary trajectories, as is the case here. Figure 4(D) shows how the relative fitness of cheaters 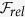 varies with cheater fraction for several different values of the ratio of fitness advantage constants Φ. We observe a unique stable fixed point for each value of Φ, that increases with Φ, suggesting that in any system where both cheating and swarming are advantageous, competing pressures can result in stable populations of cheaters that are dynamically regulated by self-organized sorting.

## Discussion

Within a group of swarming or flocking animals there may exist intrinsic behavioral heterogeneities that can effect the ability of the group to remain cohesive and move collectively. Behavioral differences can arise from natural individual variations, diseases or genetic mutations that occur randomly as the group of animals produces progeny. In artificial systems, such as robotic drones, differences can manifest as malfunctioning hardware or compromised software. In natural systems, therefore, it seems likely that there could exist collective mechanisms that allow the whole group to carry a subgroup of individuals that do not participate collective decision making (i.e non-aligners or cheaters).

We studied a system of swarming agents that display individual variations in their alignment strength. For the binary case of mixtures of aligners and non-aligners, we found that when the cohesion is strong, there is a critical fraction of cheaters f* above which collective motion of the whole group can no longer be achieved. We call this critical non-aligner fraction, the maximum non-aligner carrying capacity. We showed that there is a simple predictive relation between the maximum non-aligner carrying capacity and the length and interaction strength scales that characterize the swarm. We showed that swarms can increase their non-aligner carrying capacity f* either by increasing the separation between agents or by moving slower and/or processing information more quickly, These simple robust mechanisms could allow for a swarm or flock to adapt to increased non-aligner loads “on the fly” and our results also suggest optimal designs of parameters for “non-aligner tolerant” artificial swarms.

Alternatively, we found swarming could be enhanced in an unusual manner by lowering the cohesive attraction between agents. We showed that, as cohesion is lowered, there exists a regime where the clusters can actively sort out and leave behind the excess non-aligning population. We find that the newly sorted clusters that emerge after segregating are capable of swarming and carry a fraction of non-aligners that is near a fixed critical non-aligner carrying capacity that we are able to predict. Thus the dynamics of the swarm naturally weeds out non-aligners above the critical capacity and maintains the non-aligner fraction in the swarm close to this value, reminiscent of a self-organized critical behavior. This result highlights a simple, robust and efficient mechanism that allows heterogeneously mixed populations to naturally regulate their composition and remain in a collective swarming state despite the natural tendency for cheating/diseased/defective population fractions to increase.

Lowering the value of cohesiveness too much can also lead to deleterious effects with the swarming clusters unable to hold themselves together. This suggests that, if such a sorting and segregation mechanism is utilized in nature, the relative magnitudes of cohesiveness and orientational alignment need to be tuned to lie within an optimal range. It is also interesting to note that though we considered cheaters or defective agents as having a negative effect on swarming, one could also consider situations where a swarm needed to break up or segregate between two populations. Our results indicate that a simple mechanism to achieve this would be for one group to simply become “non-aligners” and allow the swarm dynamics to automatically segregate and expel them from the swarm.

It is to be noted that we were able to map out the entire phase space of behaviors in these heterogeneous systems including swarming, static and sorting phases and relate them to simple relations between the swarm parameters. Furthermore, these swarm parameters can be directly observed or inferred from measurements of real swarms (see Supplementary Information 4). Thus our results provide us with predictive power about real swarms including their maximum non-aligner carrying capacity, their ability to sort and the critical non-aligner fraction that sorting leads to. It is also intriguing in this context that if there is a natural tendency for non-aligners to increase, we would expect to measure, in a real swarm, a composition of non-aligners close to a critical fraction that we can predict based on the estimated swarm parameters.

We were also able to show that our notions of sorting extend to swarms with a continuous distribution of individual alignment preferences. In fact, the existence of outliers, whether they were non-aligners or “super-aligners” or both triggered the sorting process. This is suggestive of a natural means of separating different types of behavior which could have implications not only for limiting variability in populations but also more generally for the division of labor in collectively moving systems.

Finally, we pursued the idea of competing cheating and sorting by considering the evolutionary dynamics of a replicator system, where there was a fitness advantage associated with being in a collectively moving swarm and an advantage for being a cheater. We showed that the system has a globally stable evolutionary fixed point corresponding to a particular fraction of cheaters that is set by relative fitness advantage of cheating and the swarm parameters. It is interesting to note that evolutionary equilibria between successful strategies can thus be tuned by changing the physical parameters of the swarm. Our work therefore makes connections to studies of evolutionary dynamics in structured populations and networks^44^,^45^,^46^ and specifically to work that shows that both network structure^47^ and its dynamics^48^ can influence the success of cheating or defection. In the broadest sense, our results suggests the idea that the spread of a non-aligner/defector/cheater strategy within a population beyond a critical threshold can spontaneously trigger large-scale re-organization of the collective that expels outliers and effectively limits the spread of behavioral variability.

## Methods

The alignment interaction is implemented by the agent choosing an updated direction of travel that, in the absence of cohesive interactions or noise, is the sum of the direction vectors of all its nearby neighbors, which is proportional to the mean consensus direction. In our model, at each reaction time step (*δt*), agents compute the summed direction 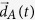 (equation (6)) of all their neighbors (nbr) within a specified “vision” distance, *d*_*v*_. *δt* is the physical reaction time of agents within the system, rather than the timestep of the simulation (Δ*t*).
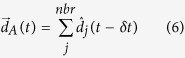


The cohesive interaction is modeled as arising from a Lennard-Jones (LJ) interaction (equation (7)) between nearest neighbor agents which is attractive at large distances and repulsive at close range. To calculate the LJ interaction, 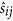 is a unit vector pointing between the *i*^*th*^ and *j*^*th*^ agents and *s* is the separation between them.
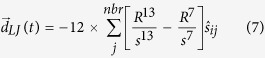


The LJ interaction is calculated as a unitless LJ direction vector, 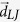. For a standard LJ potential well with depth 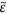 the corresponding LJ force would be 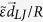. The agents calculate the alignment direction vector as well as the LJ direction vector dictated by the cohesive LJ interactions with all their neighbors (equation (7)) at each timestep. For our simulations the equilibrium separation between agents (*R* in equation (7)) is set to a distance, relative to the vision distance *d*_*v*_, such that each agent will see, on average, only their first nearest neighbors. A new direction of travel for the agent in the next time step is determined by the vector formed by adding 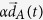 with 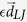, where the dimensionless parameters *α* and 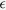 measure the importance of alignment and cohesiveness respectively (equation (1)).

New positions are then calculated for each agent according to equation (8), where *d*_*r*_ is the reaction distance, or how far an agent moves in each timestep.
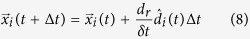


To mimic the response of real swarming individuals, we need to account for errors, either in processing local orientation information or in the execution of movement. Errors are modeled as noise that is added to the calculation of the updated direction 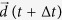 by adding a randomly selected angle into the calculated direction, 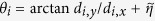, where 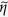 is a randomly selected angle from a uniform distribution in the interval [−*η*/2, +*η*/2]. In our model we set *η* = 0.2, *d*_*v*_ = 1, *R* = 0.7887, Δ*t* = *δt* = 1, and *α* = 1.

Swarms are generated by randomly seeding *N* agents in a circle with radius equal to the vision radius of agents, *d*_*v*_ and then allowing the swarm to set its own size by following the dynamics dictated by equation (1) and equation (8). The system has infinite boundaries and varying the initial density, and simulation time step have little effect on the resulting transitions (see Supplementary Information 1.4).

## Additional Information

**How to cite this article**: Copenhagen, K. *et al*. Self-organized sorting limits behavioral variability in swarms. *Sci. Rep.* **6**, 31808; doi: 10.1038/srep31808 (2016).

## Supplementary Material

Supplementary Information
