# Supplementary material for: Self-organized sorting limits behavioral variability in swarms
Source: Sci Rep. 2016 Aug 23;6:31808. doi: 10.1038/srep31808 (PMC4994111; doi:10.1038/srep31808)
Supplement: Supplementary Information [file srep31808-s1.pdf]

Supplementary Information

Self-organized sorting limits behavioral variability in swarms

Katherine Copenhagen David A Quint Ajay Gopinathan

1 Results and Discussion

1.1 Thermal noise driven order-disorder phase transition

Our system undergoes the typical ordered to disordered phase transition as noise is increased [35], with little system size dependence within the finite systems of the sizes we are interested in.

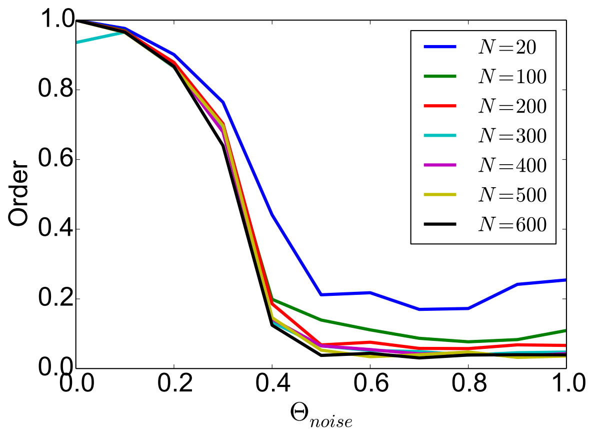

Figure S1: Order transition with noise for various system sizes.

1.2 Dependence on introduction time of non-aligners into the system

The time during the simulation when the non-aligners are introduced makes little difference to the resulting transitions as seen below. Initially the system is at $f = 0$, with no non-aligners, then at time t_0, a fraction of the system f is converted to non-aligners, and the order is measured after the system has reached steady state. Typically it takes less than 1000 time steps for the system to reach steady state, we wait until $t = 12000$ to measure the order to allow plenty of time for the system to equilibrate.

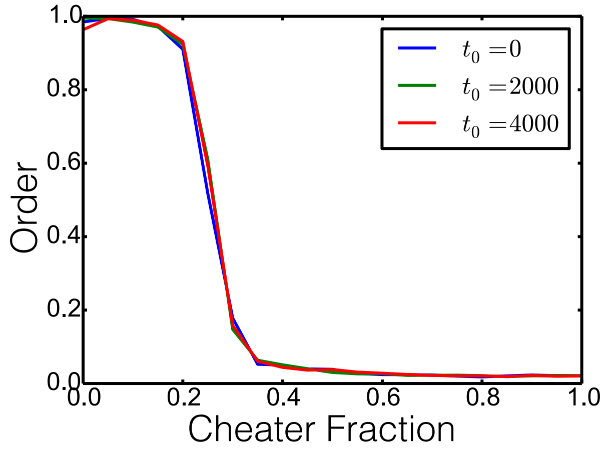

Figure S2: Order transition with non-aligner fraction where the non-aligners are introduced t_0 time steps into the simulation.

1.3 Hysteresis loop arising from introduction and removal of non-aligners

Initializing the system with all non-aligners and introducing aligners has a slightly different effect on the order of the system than vice versa. To examine the difference we measured the order of the system as non-aligners are gradually added and then removed from the system with time. Initially the system has no non-aligners and $N = 100$ aligners and then one aligner is converted to non-aligner at a time and the system is allowed to relax for 100 time steps between each agent conversion. When only one non-aligner is being added at a time it takes much less time for the system to adjust accordingly and less than 100 time steps are necessary for the system to reach steady state. When the system reaches $f = 1$ the same process is repeated in reverse, converting one non-aligner to aligner at a time, allowing 100 time steps for the system to equilibrate between each step. This results in a slight hysteresis, Fig. S3, with a lower order when non-aligners are being removed from the system rather than added.

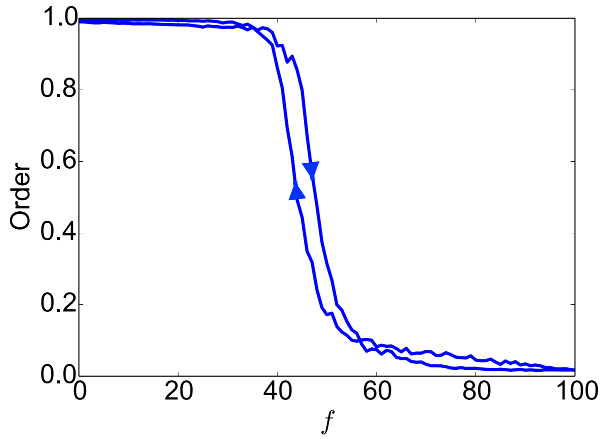

Figure S3: The order transition for the system when non-aligners are gradually added and then removed, resulting in a slight hysteresis.

1.4 Initial density and simulation timestep dependence.

Varying the initial density of agents per unit area (d_v^{-2}) has very little effect on the resulting phase transitions over a wide range as shown in Fig. S4(a). The simulation time step also has very little effect on the system dynamics as can be seen in Fig. S4(b).

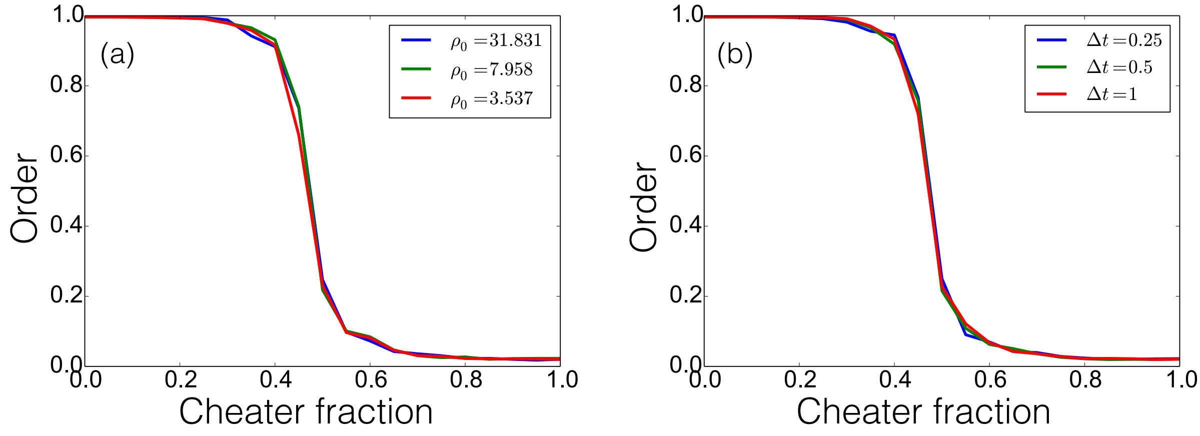

Figure S4: The order transition for the system with non-aligner fraction for varying initial system density (a), and simulation time step (b).

2 Analytic approximation for the ordered or disordered state of the system

We assume that the average magnitude of the alignment interaction on any agent chosen at random, must be greater than the average magnitude of the Lennard-Jones interaction in order for the system to be ordered, satisfying the inequality in Eq. S5.

$$\langle A \rangle_i > \langle LJ \rangle_i \tag{S5}$$

The average magnitude of the alignment interaction is the chance of an agent, chosen at random, being a aligner, $(1-f)$, multiplied by the number of agents it is interacting with, n, and the alignment interaction strength, α, as seen in the left hand side of Eq. S6. The average magnitude of the Lennard-Jones interaction is due to the fluctuations of each agent (regardless of whether it is a non-aligner or not) around the bottom of the potential well that it is in. In any time step an agent can move a distance up to d_r away from the bottom of the well, resulting in a Lennard-Jones force (of the form given in Eq. 7) at a separation of $R-d_r$ on average from each of it's n neighbors, as seen in the right hand side of Eq. S6. However, due to the fact that the n Lennard-Jones forces acting on an agent from each neighbor will point in effectively random directions, and the average magnitude of the Lennard-Jones force on the agent will be equivalent to the root mean squared distance travelled by a classic 2D random walk with n steps, resulting in the factor of $\sqrt{n}$.

$$(1-f)n\alpha > \sqrt{n}\epsilon \times 12\Big[\frac{R^{13}}{(R-d_r)^{13}} - \frac{R^7}{(R-d_r)^7}\Big] \tag{S6}$$

$$(1-f)n\alpha > \sqrt{n}\epsilon \times 12\Big[\frac{1}{(1-d_r/R)^{13}} - \frac{1}{(1-d_r/R)^7}\Big]$$

$$(1-f)n\alpha > \sqrt{n}\epsilon \times 12\Big[\Big(1+\frac{12}{1!}d_r/R+\frac{12\times 13}{2!}(d_r/R)^2+...\Big)-\Big(1+\frac{6}{1!}d_r/R+\frac{6\times 7}{2!}(d_r/R)^2+...\Big)\Big]$$

Since d_r/R is small we can drop higher-order terms.

$$\frac{\alpha(1-f)}{\epsilon} > \frac{12}{\sqrt{n}}[6d_r/R]$$

Ignoring defects and considering the mostly hexagonal lattice of agents in our system, we take the coordination number to be $n = 6$ and find

$$\frac{\alpha(1-f)}{\epsilon} > 12\sqrt{6}\frac{d_r}{R}$$

From this result we have a simple linear relationship between interaction energy scales (α, ϵ) and physical system parameters (d_r, $R(d_v)$), which we can use the predict the value of f^*,

$$f^* = 1 - \frac{12\sqrt{6}}{0.8}\frac{\epsilon}{\alpha}\frac{d_r}{d_v} \tag{S7}$$

where the average magnitude of the alignment interaction and the average magnitude of the Lennard-Jones interaction within the system become equal and the order disorder transition occurs.

3 Phase diagrams

The analytic prediction of the phases of the system holds for a wide range of parameters feasible in our simulations. Fig. S5 shows the three phases (swarming "Sw", static "St", and sorting "So") shown in the (f,ϵ) plane for three values of $d_v/d_r = 67, 100,$ and 200. The predicted transition lines are derived from Eq. S7 (solid white line), and the calculation of f^c, the critical non-aligner fraction, f^*, when $\epsilon = 1$ (dashed white line), and are accurate over a wide range of d_v/d_r as shown.

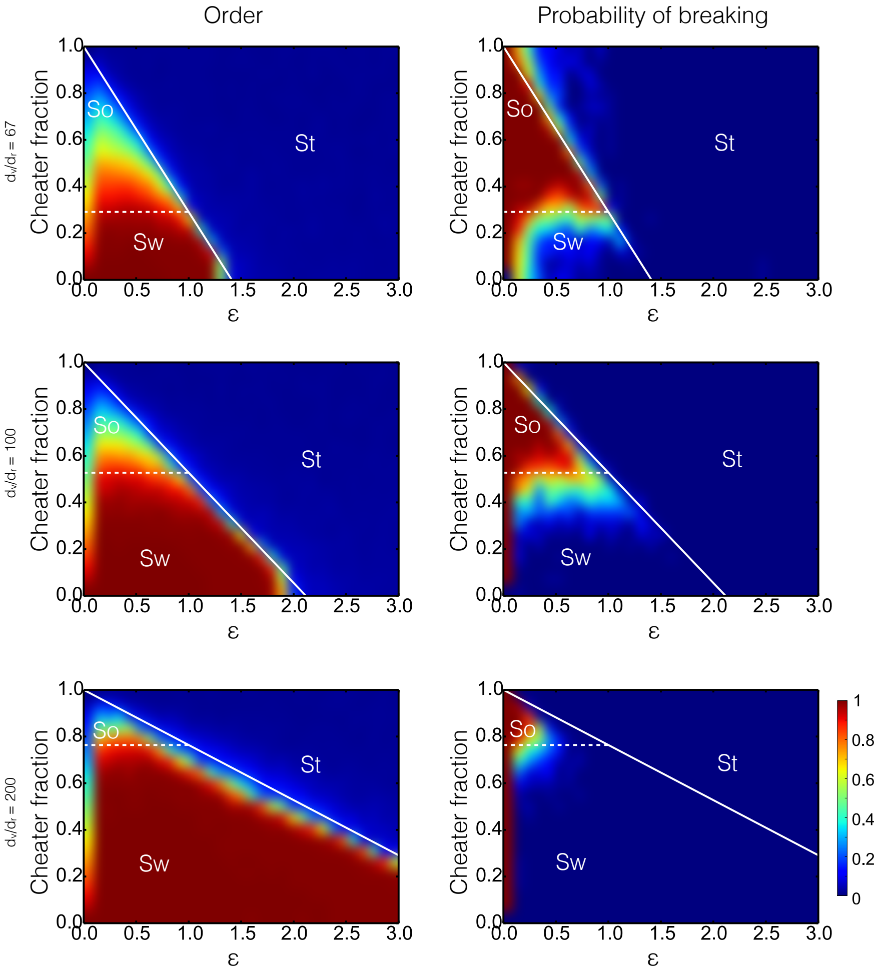

Figure S5: The phase diagram for the system with the analytically derived phase boundaries for three values of $d_v/d_r = 67, 100,$ and 200.

4 Cluster size distribution

The size distribution of clusters after sorting follow a power law as shown in Fig. S6. This case has an exponent of -1.2 however the exponent varies in a complicated way with system parameters and starting non-aligner fraction.

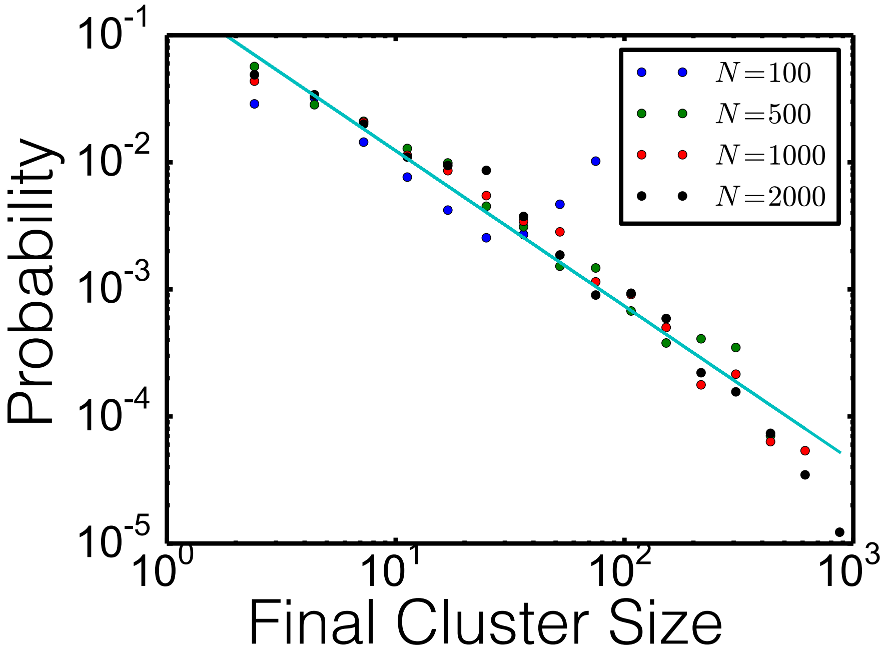

Figure S6: Size distribution of the clusters within the system after sorting has occured with $d_v/d_r = 100$ and $f^* = 0.5$.

5 Determining Interaction Strengths from Separation Measurements

The value of ϵ can be inferred by looking at how well crystalized and solid the swarm is and hence how regular the spacing between agents are. Therefore in an ordered swarm, a lower value of ϵ will result in a larger variance in agent-agent separation. In the case where ϵ is increased above the swarming threshold (see Fig. 3(a)) the dynamics of the swarm are dominated by random agent motion around the potential minimum due to the active forces exerted by agents and the relationship between ϵ and separation variance no longer holds. Fig. S7 shows the separation variance plotted against ϵ for several values of d_v/d_r, within the ordered regime, which can be directly measured in a given swarm and a clear relationship between increasing ϵ and decreasing separation variance can be seen, allowing for ϵ to be extracted from direct swarm measurements.

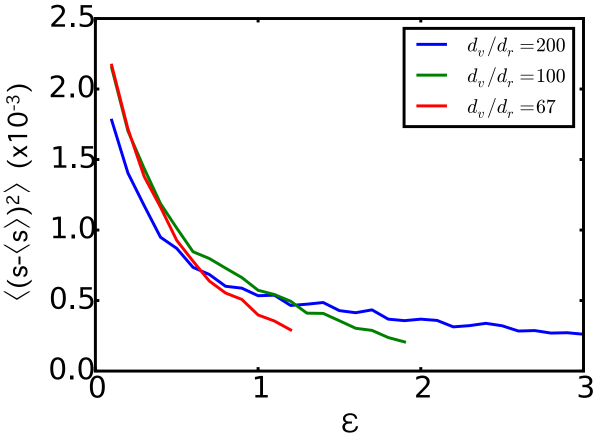

Figure S7: Variance of the separation between each agent and it's neighbors plotted against ϵ.
